# Harris Hip Score and SF-36 following metal-on-metal total hip arthroplasty and hip resurfacing - a randomized controlled trial with 5-years follow up including 75 patients

**DOI:** 10.1186/s12891-021-04671-1

**Published:** 2021-09-12

**Authors:** Peter Nyby Hersnaes, Kirill Gromov, Kristian Stahl Otte, Peter Henrik Gebuhr, Anders Troelsen

**Affiliations:** Department of Orthopaedic Surgery, Copenhagen University Hospital Hvidovre, Hvidovre, Denmark

**Keywords:** Hip replacement arthroplasty, Total hip arthroplasty, Hip resurfacing, Osteoarthritis, Harris hip score, SF-36, Metal ion concentration

## Abstract

**Background:**

The metal-on-metal large-diameter-head (MoM-LDH) hip replacements increased in popularity during the start of the twenty-first century. Subsequently reports raised concerns regarding adverse reactions due to elevated chromium (Cr) and cobalt (Co) concentrations as well as high rates of other complications and revisions.

The purpose was to compare Harris Hip Score and SF-36 at 5-years follow up following MoM-LDH total hip arthroplasty (MoM-LDH-THA) or MoM hip resurfacing (MoM-HR).

**Methods:**

The study was conducted between November 2006 to January 2012 in a tertiary health care center in Denmark. Patients with primary or secondary osteoarthritis were randomly assigned to receive a Magnum (MoM-LDH-THA) or a Recap (MoM-HR) prosthesis. Randomization was computer generated and allocation was concealed in an opaque envelope. Neither patients nor care provider were blinded. Primary outcome was Harris Hip Score at 5-years follow up.

**Results:**

Seventy-five were included and allocated to the MoM-LDH-THA (*n* = 39) and MoM-HR (*n* = 36) group. The study was prematurely stopped due to numerous reports of adverse events in patients with MoM hip replacements. Thirty-three in the MoM-LDH-THA and 25 in the MoM-HR group were available for primary outcome analysis.

Median Harris Hip Score was 100 (IQR: 98–100) for MoM-LDH-THA and 100 (IQR: 93–100) for MoM-HR (*p* = 0.486). SF-36 score was high in both groups with no significant difference between groups.

**Conclusion:**

Harris Hip Score and SF-36 score was excellent in both groups with no significant difference at 5-years follow up. Our findings suggest that there is no clinical important difference between the two prostheses implanted 5 years after implantation.

**Trial registration:**

ClinicalTrials.gov, NCT04585022, Registered 23 September 2020 – Retrospectively registered.

This study was not prospectively registered in a clinical trial database since it was not an entirely implemented standard procedure in the international orthopedic society when the study was planned.

## Background

During the start of the twenty-first century the use of metal-on-metal large-diameter-head total hip arthroplasty (MoM-LDH-THA) and metal-on-metal hip resurfacing (MoM-HR) increased rapidly. Proposed advantages over the conventional small-head metal-on-polyethylene articulation were low wear of implant, increased range of motion and reduced dislocation rates [1–4]. Subsequently reports in the 2010s raised concerns regarding association between elevated metal ion concentration in blood and locally as well as systemic complications in patients with MoM hip implants [5–7]. The use of MoM implants declined worldwide after international regulatory agencies issued alerts and safety communications related to the use of MoM hip implants [8–10]. Local complications due to metal wear from MoM hip implants are broadly referred to as adverse reaction to metal debris (ARMD) and includes joint failures associated with pain, large sterile effusion of the hip and macroscopic metallosis [11].

It has been estimated that over one million patients worldwide have received a MoM hip implant [12]. Identifying risk factors associated with high metal ion concentrations in patients who have received a MoM hip implant as well as whether or not there is a medium to long term clinical difference between MoM implant designs is therefore of great importance.

We aimed to investigate the possible differences in Harris Hip Score and SF-36 in patients following single brand MoM-HR or single brand MoM-LDH-THA as well as radiological findings, metal ion concentrations and rate of revision at 5-years follow up.

We hypothesize that there is no clinical difference in Harris Hip Score and SF-36 when comparing patients receiving MoM-LDH-THA and MoM-HR 5 years after primary surgery.

## Methods

### Study design

This was a single center parallel-group randomized controlled trial. We compared outcome between groups at 5-years follow up.

Perceived hip function was the primary outcome measured with Harris Hip Score at 5-years follow up.

Secondary outcomes were SF-36, metal ion concentrations (Cr and Co), radiological findings and revision rate at 5-years follow up. The study was planned and started before the association between elevated blood metal ions and complications in patients with MoM hip implants was known. The study was prematurely terminated in 2012 due to alerts and safety communications from the Danish national regulatory agency regarding early failure rates and high incidence of ARMD in patients with MoM hip implants [13]. At that time the study population consisted of 75 patients out of 200 planned for inclusion.

### Subjects

The study was conducted in a tertiary health care center in Copenhagen, Denmark from November 2006 to January 2012. 5-years follow up consultations were conducted from 2011 to 2017. Eligible patients were men aged 18 to 70 and women aged 18 to 65 suffering from primary or secondary hip osteoarthritis eligible for hip replacement surgery according to guidelines at the department at that time. Understanding and speaking Danish, able to give informed consent and able to complete follow up consultations were obligatory.Exclusion criteria were earlier or present infection of the hip, severe systemic or metabolic disease leading to weakening of the bone, severe congenital hip dysplasia, osteoporosis and renal disease.

### Randomization and allocation

All patients were randomly allocated to receive either a Magnum (Zimmer Biomet, Warsaw, IN) or a Recap (Zimmer Biomet, Warsaw, IN) implant. A computer program was used for randomly generating an allocation sequence and allocation was concealed in an opaque envelope until the day of surgery. Neither patients nor care provider were blinded. The laboratory analyzing blood metal concentrations were blinded to treatment allocation throughout the study.

### Surgical procedure and recovery

**Fig. 1:**
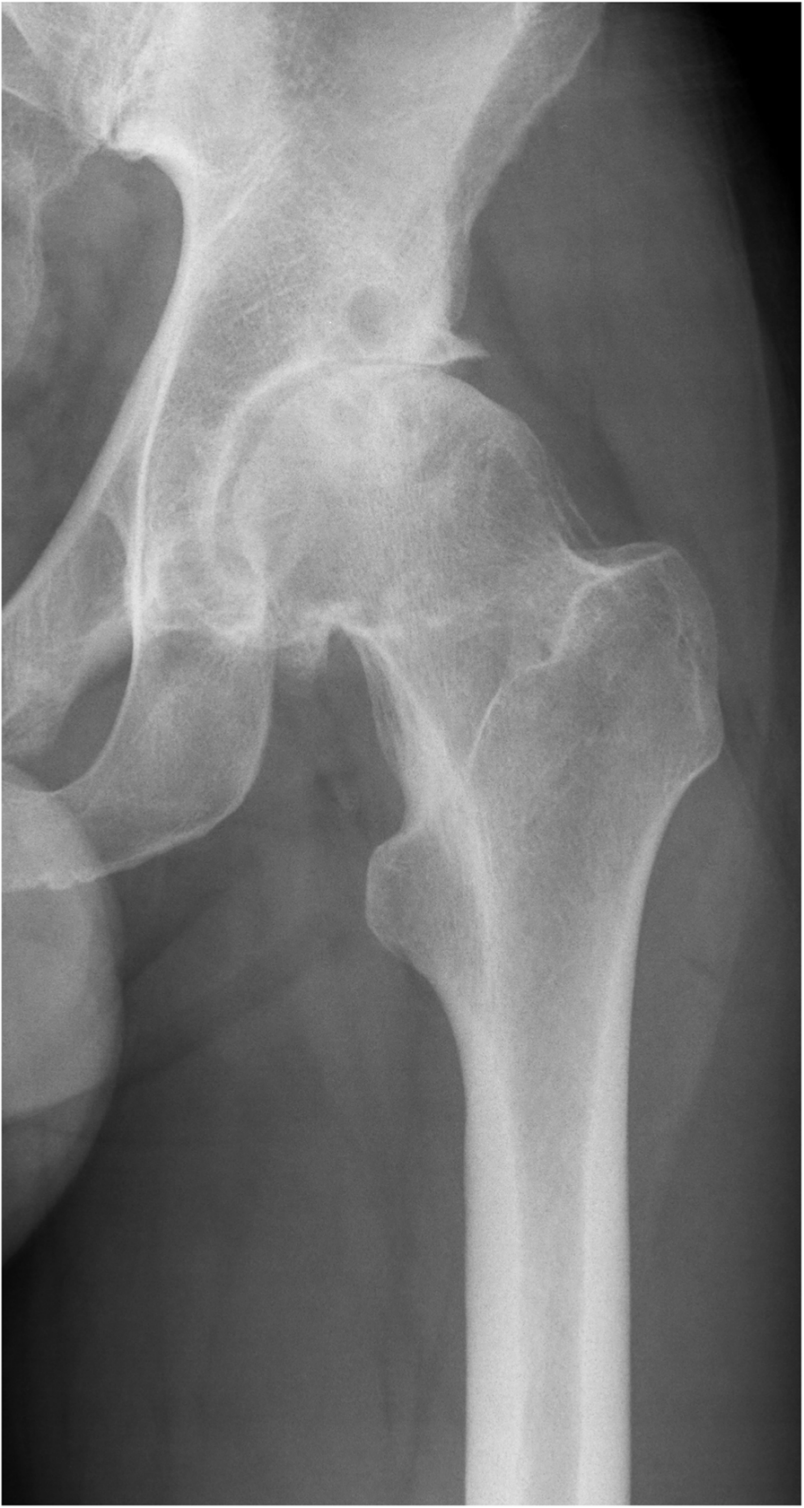
Preoperative x-ray of severe osteoarthritis

**Fig. 2:**
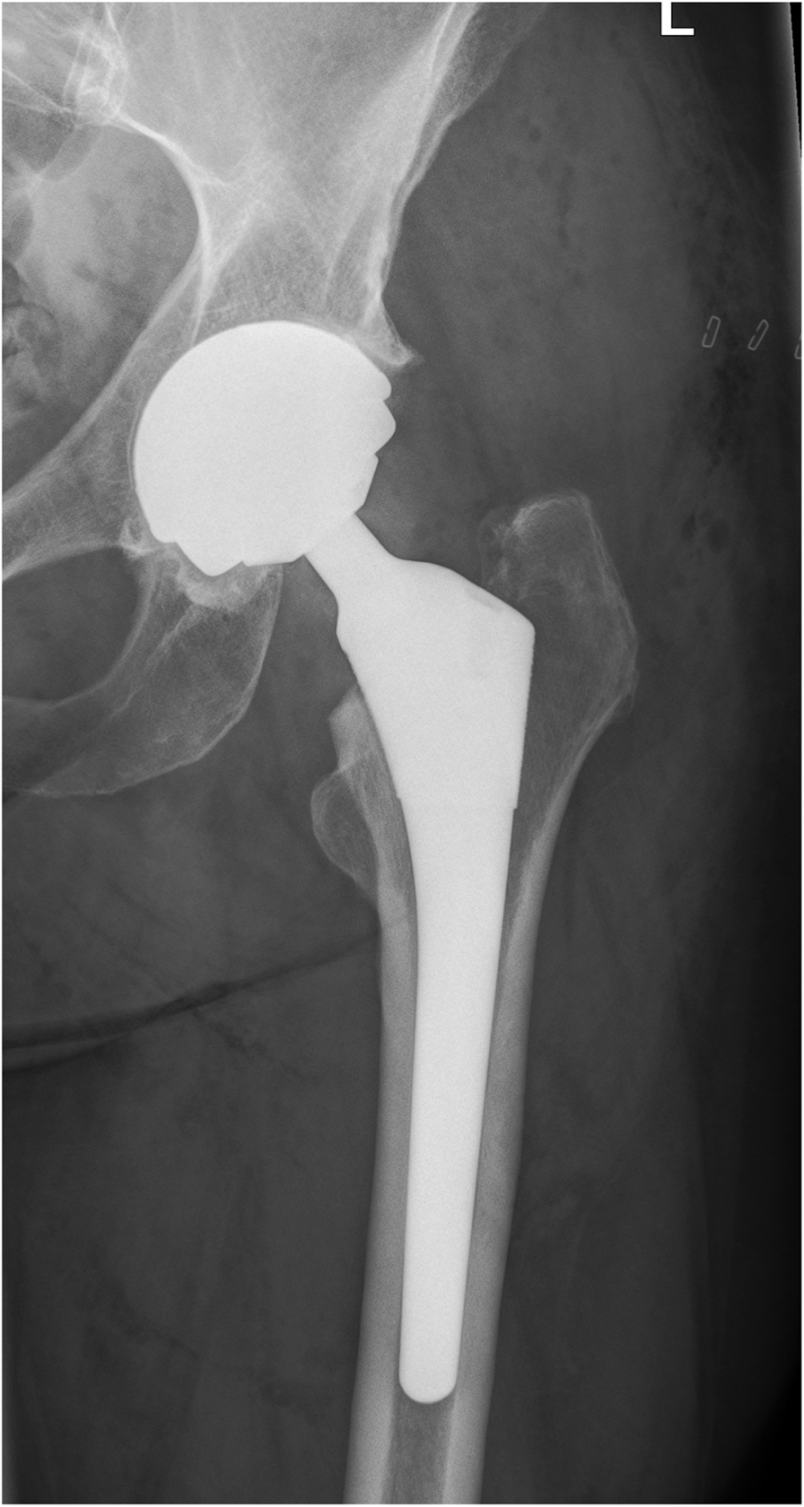
Postoperative x-ray (MoM-LDH-THA)

**Fig. 3:**
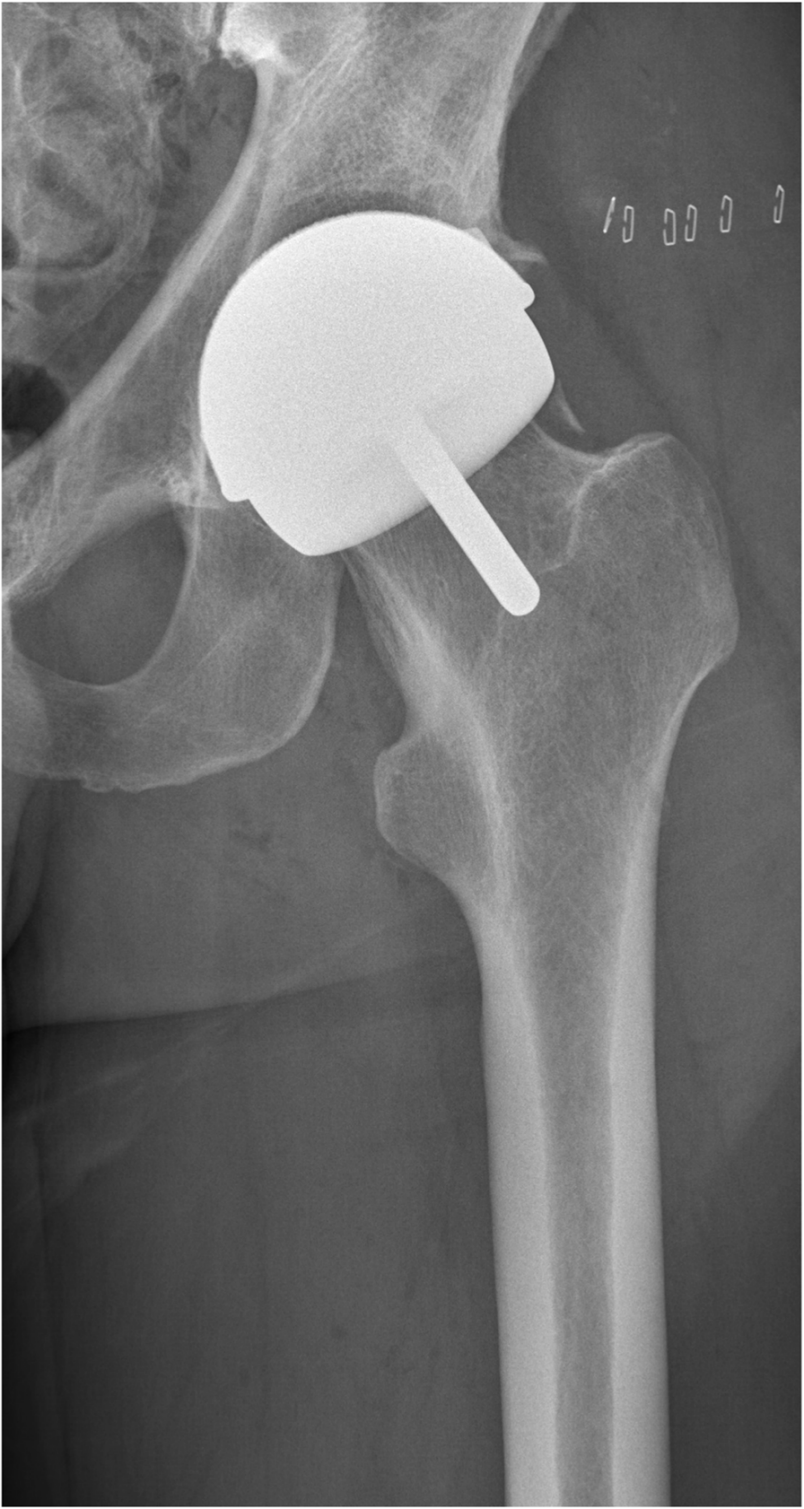
Postoperative x-ray (MoM-HR)

Two experienced surgeons performed the surgeries through a standard posterolateral approach in both groups. The MoM-LDH-THA prosthesis included the uncemented Bimetric Hip Primary Femoral Porous coated collarless stem, the M2a Magnum Taper Adaptor and the M2a Magnum Modular Head. The MoM-HR prosthesis included the cemented (Refobacin Bone Cement, Zimmer Biomet, Warsaw, IN) Recap Resurfacing System Femoral Head. The uncemented Recap/Magnum Acetabular Shell was used in both groups. Pre- and postoperative x-ray images are seen in Fig. 1 (preoperative), Fig. 2 (MoM-LDH-THA) and Fig. 3 (MoM-HR). The standard techniques for insertion as imposed by the manufacturer was followed. Both groups received the same standardized thromboprophylactic and prophylactic antibiotic treatment. The two groups followed the same standardized postoperative rehabilitation plan allowing immediate weight bearing as tolerated and physiotherapy starting from day of surgery and continued after discharge as outpatient rehabilitation.

### Outcome evaluation

#### Patient reported outcome measure

Harris Hip Score was used for measuring hip function. Scoring was based on the instruction by Mahomed et al. [14] with the following alterations : 1) The question addressing public transportation was included and 2) All participants received 4 points indicating no fixed deformity or limb length discrepancy of 3.2 cm or more.

SF-36 version 1 was used for measuring quality of life. Scoring was based on the original manual “SF-36 Health Survey Manual & Interpretation Guide” [15]. Median values were used when interpreting PROM scores. Score range was 0-100 for both questionnaires with high scores indicating good perceived hip function and quality of life respectively.

#### Metal ion measurement

Chromium and cobalt concentrations were measured in whole blood using an inductively coupled plasma sector field mass spectrometer (ICP-SFMS) on the Thermo Fischer ELEMENT 2 (Thermo Fisher Scientific Inc. Waltham, MA).

Patients with bilateral MoM hip implants at 5-years follow up were excluded from metal ion analysis.

#### Radiological analyses

##### Radiolucency and osteolysis

Radiological analysis for radiolucency and osteolysis was done with the software mDESK™ version 3.6.7.0 (RSA Biomedical, Umeaa, Sweden). The acetabular component in both groups was analyzed for radiolucency as described by DeLee and Charnley [16]. In the MoM-LDH-THA group the stem was analyzed for radiolucency as described by Gruen et al. [17]. Radiolucency > 2 mm was considered pathological. All radiological analyses were performed by an orthopedic surgeon under training. In case of doubt consensus was achieved after consulting with a senior colleague.

##### Inclination angle and version

Cup inclination angle and degree of anteversion were measured using Martell’s Hip Analysis Suite version 8.0.4.3 (University of Chicago, Chicaco, IL). Analyses were conducted on calibrated anteroposterior pelvic radiographs. In hips with an estimated anteversion equal or lesser than 10 degrees (*n* = 29) a shoot-through lateral radiograph was analyzed for ante- or retroversion as done by Callanan et al. [18].

#### Implant survival

Patients undergoing revision before 5-years follow up were identified. Date as well as reason for revision were registered. Cross-sectional imaging was performed when indicated in accordance to the national guidelines from the Danish Orthopaedic Society [19].

### Statistical analysis

All statistical analyses were performed using the statistical software RStudio version 1.0.153 (RStudio, Inc., Boston, MA, URL http://www.rstudio.com). Comparisons between groups were performed using Wilcoxon Rank Sum test for non-parametric numerical data and Pearson’s Chi-squared test for non-parametric ordinal data. A *p*-value < 0.05 was considered significant.

### Power analysis

The original sample size calculation estimated that 100 patients in each group would be needed to detect a 3-point difference between groups in a 2-sided significance test with a power of 0.8 and an alpha error level of 0.05 when assuming a standard deviation of 7 points in Harris Hip Score and a dropout rate of 15%.

### CONSORT statement

This study adheres to CONSORT guidelines.

## Results

### Baseline demographics

Total median age was 61.9 years in the MoM-LDH-THA group and 59.4 years in the MoM-HR group. Gender ratio (M/F) was 2.00 in the MoM-LDH-THA group and 2.60 in the MoM-HR group. All baseline demographics are shown in Table 1.

**Table 1:** Baseline demographics

		Total	MoM-LDH-THA	MoM-HR	
		*n* = 75	*n* = 39	*n* = 36	
		(1st – 3rd quartile)	(1st – 3rd quartile)	(1st – 3rd quartile)	*p*-value
Median age	Total	60.8 (54.55–63.3)	61.9 (56.5–63.2)	59.4 (51.2–63.58)	0.107
	Males	61.95 (55.62–64.83)	62.65 (60.65–64.88)	60.35 (53.60–64.40)	0.253
	Females	58.40 (53.00–61.00)	59.40 (55.40–62.30)	53.00 (50.92–59.75)	0.088
Gender ratio (M/F)		2.26	2.00	2.60	0.79
Median BMI		27.9 (25.2–31.0)	28.4 (25.8–28.93)	27.45 (24.6–29.32)	0.12

### Patient flow

**Fig. 4:**
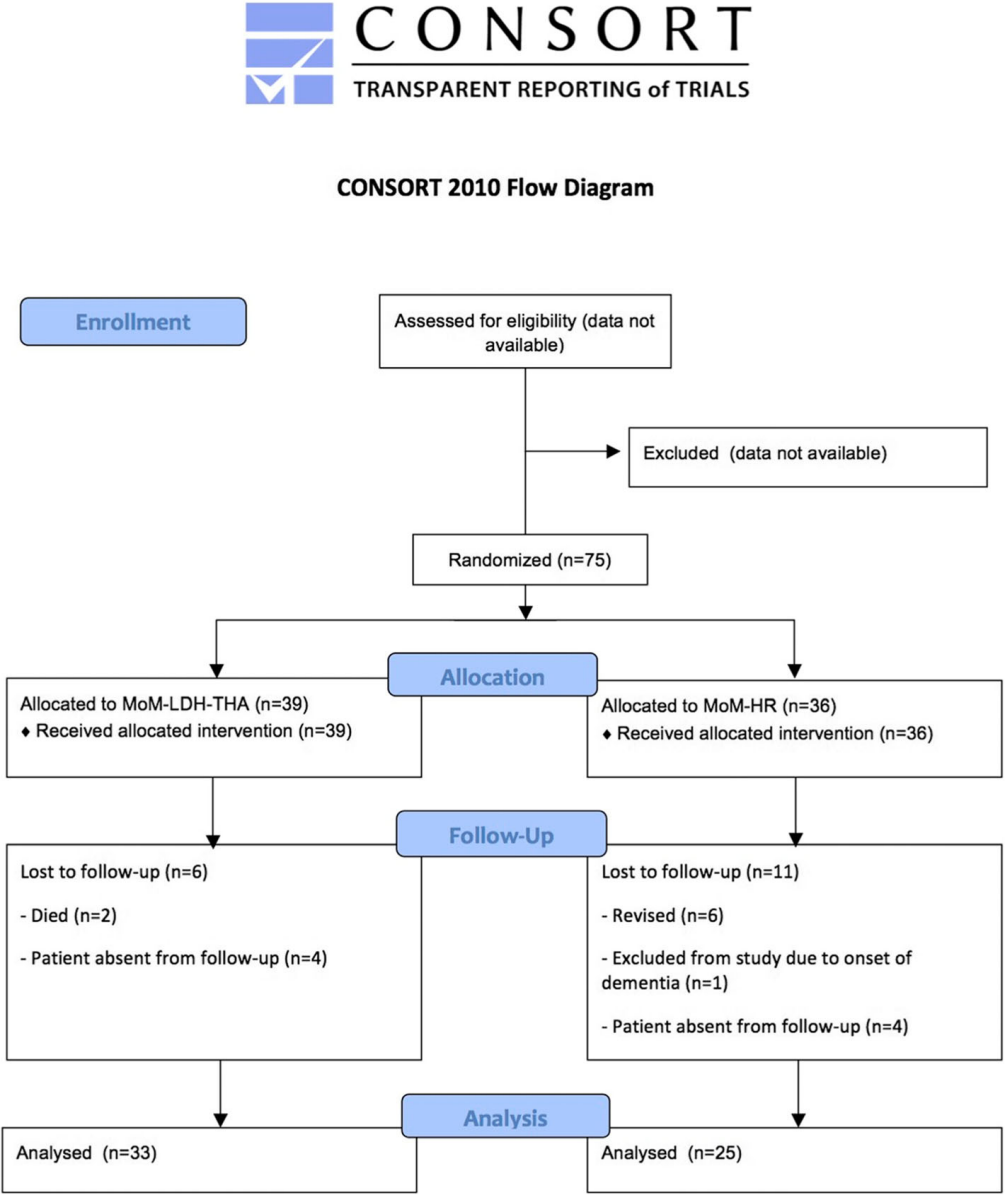
Consort diagram illustrating flow of patients during enrollment, allocation, follow up and analysis

A CONSORT Flow Diagram is presented in Fig. 4. Regarding secondary outcomes: 25 patients in the MoM-LDH-THA group and 20 patients in the MoM-HR group were available for metal ion concentration analysis at 5-years follow up excluding bilateral MoM hip replacement, lost to follow up, revision or died before 5-years follow up.

### Head size

Median (IQR) head diameter was 48 (46–50) and 50 (47.5–52) in the MoM-LDH-THA and MoM-HR group respectively (*p* = 0.13).

### Cup inclination and version

Median cup inclination angle was 40.94° IQR (35.28°-47.06°) in the MoM-LDH-THA group and 43.05° IQR (35.91°-46.06°) in the MoM-HR group (*p* = 0.94). Median cup anteversion was 12.24° IQR (5.79°-17.69°) in the MoM-LDH-THA group and 12.34° IQR (5.35°-16.89°) in the MoM-HR group (*p* = 0.84).

### Patient reported outcome measures

Both median Harris Hip Score and median SF-36 subset scores were high in the two groups. No significant differences in Harris Hip Score or SF-36 subset scores were found between the two groups (Table 2). No baseline data were obtained since the aim of the study was to compare scores between groups at 5 years follow up and not to compare paired data. We are confident that the randomization design of the study protects against any significant difference in preoperative scores between groups.

**Table 2:** Patient reported outcome measure scores at 5-years follow up

		Total (1st – 3rd quartile)	MoM-LDH-THA (1st – 3rd quartile)	MoM-HR (1st – 3rd quartile)	*p*-value
Median Harris Hip Score		100 (96–100) *n* = 58	100 (98–100) *n* = 33	100 (93–100) *n* = 25	0.486
Median SF-36 Subset Score	Physical function	90 (70–95) *n* = 53	90 (68.75–95) *n* = 28	90 (70–100) *n* = 25	0.843
	Physical restriction	100 (66.67–100) *n* = 53	100 (72.92–100) *n* = 28	100 (50–100) *n* = 25	0.512
	Bodily pain	84 (62–100) *n* = 56	92 (62–100) *n* = 30	84 (62–100) *n* = 26	0.629
	General health	77 (57–87.75) *n* = 52	74.5 (61.75–89.25) *n* = 26	79.5 (56.44–87.00) *n* = 26	0.57
	Vitality	75 (50–85) *n* = 54	77.5 (60–85) *n* = 28	75 (32.5–85) *n* = 26	0.674
	Social function	100 (100–100) *n* = 55	100 (100–100) *n* = 29	100 (100–100) *n* = 26	0.991
	Emotional restriction	100 (100–100) *n* = 53	100 (100–100) *n* = 28	100 (100–100) *n* = 25	0.411
	Mental Health	90 (72–96) *n* = 52	92 (72–96) *n* = 27	88 (76–92) *n* = 25	0.4

### Cobalt and chromium concentrations

Median chromium concentration was 1.36 μg/L in the MoM-LDH-THA group and 1.21 μg/L in the MoM-HR (*p* = 0.46). Median cobalt concentration was 1.67 μg/L in the MoM-LDH-THA group and 0.92 μg/L in the MoM-HR group (*p* = 0.073) (Table 3).

**Table 3:** Whole blood metal ion concentrations at 5-years follow up

		Total (1st – 3rd quartile)	MoM-LDH-THA (1st – 3rd quartile)	MoM-HR (1st – 3rd quartile)	*p*-value
Median whole blood metal ion concentrations (μg/L)	Chromium	1.35 (0.96–3.11) *n* = 45	1.36 (0.99–3.11) *n* = 25	1.21 (0.88–3.03) *n* = 20	0.46
	Cobalt	1.11 (0.69–2.15) *n* = 45	1.67 (0.86–2.31) *n* = 25	0.92 (0.64–1.49) *n* = 20	0.073

The lower detection limit for metal ion concentration was 0.5 μg/L. In three cases, all in the MoM-HR group, chromium concentrations were below this limit and an estimated concentration of 0.25 μg/L was used.

The very first blood analysis determined cobalt and chromium concentrations to be < 7.0 μg/L with no further specification. The analyses were made on a different instrument than all the subsequent blood analyses and were excluded. This incident only lead to exclusion of 1 patient from metal ion concentration analysis with limited impact on estimates.

### Radiological findings

No radiolucency was found in any patient. One case of osteolysis was seen in the MoM-HR group 2 years after surgery and revision was done. Revision surgery was done and septic loosening was diagnosed after cultivation of *Staphylococcus epidermidis* in 4 out of 6 tissue samples.

### 5-years revision rates

5-years revision rate was 0% in the MoM-LDH-THA group and 16.7% (*n* = 6) in the MoM-HR group. The causes for revision were facture of the femoral neck (*n* = 2), aseptic loosening of cup (*n* = 1), unexplained hip pain without joint failure (*n* = 1), ARMD (*n* = 1) and septic loosening (*n* = 1).

## Discussion

Both the MoM-LDH-THA and the MoM-HR group showed excellent PROM scores at 5-years follow up. Median SF-36 subset scores was equal or even better compared to the Danish background population [20]. Neither Harris Hip Scores nor any SF-36 subset scores varied significantly between the two groups.

In this report of a prematurely terminated randomized controlled trial with 75 patients included we assessed and compared Harris Hip Score and SF-36 between a single brand MoM-LDH-THA (*n* = 33) and a single brand MoM-HR (*n* = 25) as well as whole blood metal ion concentrations, radiological findings and revision rate at 5-years follow up.

Similar to our study Borgwardt et al. [21] found no significant difference between the Magnum and the Recap group in Harris Hip Score at 5-years follow up. Regarding SF-36 no 5-years follow up results were reported but a significantly lower score in 3 out of 8 subsets were reported at 7-years follow up in the Recap group compared to the Magnum and the ceramic-on-ceramic group.

Costa et al. [22] conducted a randomized controlled trial with 122 patients. All HR implants had MoM bearings while bearing material in the THA group included both ceramic-on-ceramic, ceramic-on-metal and metal-on-metal. No significant difference in Harris Hip Score between HR and THA was found 1 year after surgery.

Garbuz et al. [23] conducted a randomized controlled trial with 104 patients receiving either the Durom resurfacing component (*n* = 48) or the M/L Taper stem (*n* = 56). Both groups received the same Durom acetabular cup. Similar to Costa et al. no difference was shown regarding SF-36 between groups 1 year after surgery.

We found raised whole blood metal ion concentrations in both groups with a close to significantly higher median cobalt concentration in the MoM-LDH-THA group (1.67 μg/L) compared to the MoM-HR group (0.92 μg/L) (*p* = 0.073). No significant difference in median chromium concentration between the MoM-LDH-THA group (1.36 μg/L) and the MoM-HR group (1.21 μg/L) was found (*p* = 0.46). Despite raised metal ion concentrations was found in both groups we do not suspect concentrations in this range to be of any clinical significance in either of the groups. However we believe that these findings indicate a need for longer than 5 years follow up period in terms of measuring blood metal ion concentration especially in patients with MoM-LDH-THA.

Intravascular metal ion concentrations are measured in either serum or whole blood. Absolute values of chromium and cobalt in serum and whole blood are not comparable but it should be noted that intrapersonal cobalt and chromium concentrations are higher in serum compared to whole blood [24].

To our knowledge only a few studies comparing metal ion concentrations between MoM-LDH-THA and MoM-HR at 5-years follow up or longer exist.

In a randomized controlled trial [21] comparing the Magnum (*n* = 36), Recap (*n* = 41) and a ceramic-on-ceramic (*n* = 49) implant significantly higher serum cobalt concentrations were found in the Magnum group (median 2.10 μg/L) compared to the Recap group (median 1.12 μg/L) at 5-years follow up (*p* = 0.029). Concurrently no differences in serum chromium concentrations were found.

In a systematic review [25] on Cr and Co concentrations in multiple MoM implants (both THA and HR) a peak concentration was calculated after a minimum of 1-year follow up. Median whole blood Co values ranged between 0.7 and 2.7 μg/L while median whole blood Cr values ranged between 0.5 and 2.5 μg/L. Our results from both the MoM-LDH-THA and MoM-HR group for both median Co and Cr values lies in the mid part of the ranges. It should be noted that our median values are from one point in time while the systematic review reported maximum metal ion concentrations from different points in time.

In a retrospective comparative study [26] of 77 well functioning Birmingham MoM-HRs and 42 well functioning Birmingham MoM-LDH-THAs both paired with the same BHR cup a significantly higher mean serum cobalt concentration at a mean follow up time of 39.3 months was found in the MoM-LDH-THA group (2.75 μg/L) compared to the MoM-HR group (1.52 μg/L) (*p* < 0.001). Similar to our study no significant difference in chromium concentrations was found in the study.

Taking the above into account it seems that both the MoM-LDH-THA and MoM-HR implant used in our study are in the lower range regarding metal ion concentrations at mid-term follow up when comparing with other brands with similar design. Additionally, Hutt et al. [27] found the lowest whole blood cobalt and chromium concentrations in the Magnum implant when comparing with three other types of MoM-LDH-THA (Durom, Birmingham, ASR XL) at 5-years follow up with differences between Magnum and Durom or Birmingham being significant.

There is a difference regarding wear and corrosion between MoM-LDH-THA and MoM-HR. While both implant types have identical MoM bearings it is well known that the head-neck junction in the MoM-THA is an additional source of metal ion release [28]. In LDH-THA a trunnion is often used to connect the head and neck resulting in trunnion corrosion leading to additional release of metal ions. A recent systematic review [29] revealed higher rate of trunnion corrosion in mixed metal alloys at the head-neck junction compared to head-neck junctions with similar metal alloys due to galvanic corrosion. The trunnion in the MoM-LDH-THA implant in our study was made of a titanium alloy.

The 5-years revision rate was 0% in the MoM-LDH-THA group and 16.67% (*n* = 6) in the MoM-HR group. One revision (ARMD) was related to the concept of MoM articulation while two revisions (fracture of the femoral neck) were related to HR design. Our findings are not similar to previous studies findings.

In the before mentioned randomized controlled trial comparing Magnum, Recap and a ceramic-on-ceramic implant [30] 7-years revision rate was 3.9% (*n* = 2) in the Recap and 4.3% (*n* = 2) in the Magnum group.

In a Scandinavian register study [31] (*n* = 32,678) they compared midterm revision rates between THA with either MoM or metal-on-polyethylene (MoP) bearings as well as revision rates between different MoM-THA designs. The cumulative incidence of revision in the MoM-THA with the ASR acetabular component was more than 25% at 5.8 years. When excluding the ASR acetabular component they found an 8-years revision risk of 5.0% in the MoM-THA. When comparing specific cup and stem combinations a significantly higher RR of revision was seen in the M2a/Bi-metric, ASR/Summit and ASR/Corail compared to the Recap/Bi-metric combination. No difference in RR between MoM-THA and MoP-THA was found when excluding the ASR acetabular components. To some extend this could explain why the MoM-LDH-THA group in our study, which had a Recap acetabular component, had a very low 5-years rate of revision.

A Finnish nationwide register study [32] reported significantly higher revision risk in the ASR/Corail THA compared to the ASR HR (RR = 0.73, *p* = 0.04) while no significant revision risk was found between the Recap/Bi-metric THA and Recap HR or the Synergy/BHR THA and BHR resurfacing.

In a registry report [33] comparing revision rates for Birmingham MoM-HR (*n* = 8453) and Birmingham MoM-LDH-THA (*n* = 2101) 5-years revision rate was 3.2% in the MoM-HR group and 4.9% in the MoM-LDH-THA group with no significant difference between groups.

Despite the lack of power it is noticeable that the revision rate in the MoM-HR group in our study was higher than in all the studies mentioned while the revision rate in the MoM-LDH-THA group was lower than in all the studies mentioned.

Our study has some limitations. Due to a revision rate of 16.7% in the MoM-HR group and lost at follow up (15.4 and 13.9% for MoM-LDH-THA and MoM-HR respectively) in both groups data were not complete. No preoperative data were obtained for our primary or secondary outcomes. This is a limitation in means of data comparison between groups postoperatively. However we consider it safe to assume that no preoperative difference in Harris Hip score or SF-36 were present considering that there was no significant difference in baseline demographics and due to the randomization design of the study. We also consider it safe to assume that no preoperative difference in whole blood metal ion concentrations were present between the two groups as we excluded patients who had a contralateral MoM hip implant at day of surgery. One must assume that patients without a MoM articulating hip implant have very low amounts of cobalt and chromium in the blood. Our findings could not demonstrate any significant difference in Harris Hip Score nor SF-36 subset scores. However due to premature termination of the study it lacks power causing a risk of type II error. Therefore our findings must be interpreted with caution.

Despite the lack of power our results demonstrate a tendency towards higher metal ion concentrations in the MoM-LDH-THA group compared to the MoM-HR group which is in accordance to other studies findings [26, 30].

## Conclusion

Harris Hip Score and SF-36 scores were excellent in both groups with no significant difference at 5-years follow up. Our findings suggest that there is no clinical important difference between the two prostheses implanted 5 years after implantation. However due to lack of power our findings must be interpreted with caution. Whole blood metal ion concentrations were similar following MoM-LDH-THA and MoM-HR implantation at 5-years follow up. Cobalt concentration was close to being significantly higher in the MoM-LDH-THA group while no significant difference was found in chromium concentrations between the two groups. We found a high 5-year revision rate of 16.7% in the MoM-HR group and a low 5-year revision rate of 0% in the MoM-LDH-THA.

## Data Availability

The datasets used and analyzed during the current study are available from the corresponding author on reasonable request.

## Abbreviations

MoM-LDH: Metal-on-metal large diameter head
Cr: Chromium
Co: Cobalt
THA: Total hip arthroplasty
HR: Hip resurfacing
IQR: Interquartile range
ARMD: Adverse reaction to metal debris
ICP-SFMS: Inductively coupled plasma sector field mass spectrometer
PROM: Patient reported outcome measure
